# Dialogues in colour and behaviour: integration of complex signalling traits and physiology

**DOI:** 10.1098/rspb.2025.0118

**Published:** 2025-06-25

**Authors:** Subhasmita Patro, Thejaswini Saravanan, Ayush Parag, Maria Thaker

**Affiliations:** ^1^ Centre for Ecological Sciences, Indian Institute of Science, Bangalore, Karnataka 560012, India; ^2^ School of Biological Sciences, National Institute of Science Education and Research, Bhubaneswar, Odisha 752050, India

**Keywords:** animal communication, multi-component signals, redundancy, multiple messages, steroid hormones, lizard

## Abstract

Animal communication is complex, involving multiple static and dynamic traits that vary in redundancy. To understand how multiple signals are integrated and maintained, we staged male–male interactions between size-matched individuals of *Psammophilus dorsalis* and measured display behaviours, the maximum chromatic contrast of dynamically changing skin colours and size of ultraviolet patches. We also measured testosterone and corticosterone levels induced by the social interaction. All signalling traits condensed in a principal component analysis into two main components—PC1, comprising behaviours (headbobs and shudders) and ultraviolet patch size, and PC2, representing the dorsal and lateral body colours. Testosterone levels were positively associated with PC1, and both testosterone and corticosterone levels were negatively associated with PC2. Our results suggest that the chromatic contrasts of colours are non-redundant with the intensity of behaviours, and that size of UV patches better reflects behavioural intensity than other colour components. We also found that during social interactions, individuals matched the intensity of display behaviours and the contrast of their dorsal (but not lateral) colours with their opponents. Overall, these results highlight how multiple dynamic signals, which change in response to opponents during social interactions, can be maintained both by internal mechanisms (i.e. hormones) and through non-redundancy.

## 1. Introduction

Social interactions in animals are often multi-faceted and complex. Such interactions can involve elaborate signalling mediated by a combination of behavioural, morphological and physiological traits that interact in complex feedback loops. While beneficial for communication, maintaining multiple signals can be energetically costly [1,2], and, if conspicuous to predators or inter-specific competitors, can incur ecological costs to survival [3,4]. Despite their potential costs, the widespread usage of multi-component signals during social interactions raises important questions about how such signals are maintained and the functional and physiological mechanisms underlying their maintenance.

The evolution and maintenance of multi-component signals in animal communication have been explained using several broad frameworks of hypotheses. These are based on transmission efficacy, information content, and receiver psychology [5,6,7]. Within the content-based framework, multiple signals that contain redundant information about the signaller’s quality (redundant signal hypothesis) are expected to be strongly correlated. On the other hand, when different signals convey distinct pieces of information (multiple messages hypothesis), we do not expect the same level of associations among them. Thus, examining the degree of association between multiple signals can provide insights into their roles as either redundant or non-redundant signals [7].

The complexity of social interactions is further augmented by multicomponent signals that can vary across time and space. Based on their temporal expression patterns, signals can be classified as static or dynamic. Behaviour expressed during social interactions is a clear example of dynamic signalling [8,9]. Many species can also dynamically change skin colours and patterns to communicate [10]. Dynamic signals that can change during an interaction reflect current conditions and can be adjusted by the signaller depending on their internal state as well as receiver responses. This flexibility creates opportunities for cheating as animals can modulate signal intensity to dishonestly portray higher quality, thereby influencing interaction outcomes. However, receiver responses can help maintain signal honesty by imposing social costs on dishonest individuals [11]. For instance, dishonest signallers may face increased aggression, deterring lower-quality individuals from cheating. Thus, receiver responses can play a crucial role in the evolution and maintenance of dynamic multi-component signals.

Signals of the same type may be causally linked due to common underlying mechanisms. For example, multipe colours expressed by an animal can originate from the same pathway. Similarity in structure or mode of production can lead to the encoding of similar information about the signaller (shared pathway hypothesis—index hypothesis [12]). On the other hand, resource limitations may induce trade-offs between signals, resulting in expression of one signal at the expense of another (resource trade-off hypothesis [12,13]). In varied tits (*Sittiparus varius*), carotenoid plumage coloration and tail length are negatively correlated, suggesting energetic trade-offs [14]. Dynamic changes in skin colours on reptiles, amphibians and cephalopods are imparted by movements of pigment molecules within a chromatophore unit (reviewed in [15]). Since they share the same biochemical pathways during signal expression, multiple colours might potentially encode redundant messages. On the other hand, different colour pigments such as carotenoids, melanin and structural UV colours are sourced and produced by different mechanisms in the body [16–18] and hence might reflect different types of information about the signaller’s foraging efficiency, immunocompetence or dominance. Similarly, different behavioural displays require agile movement of body parts, involving muscles that need to endure high energy demands [19]. The intensity of each type of behavioural display might therefore be limited by the motor capabilities of the relevant limb region. Thus, regardless of whether signals serve to attract attention or convey information, components of signals may be intrinsically linked because of their mechanism of expression.

In many species, social interactions involve a change in steroid hormone levels that can modulate signal expression. Numerous studies on rodents, lizards and birds have established the role of testosterone in facilitating heightened aggressive behaviour and the expression of secondary sexual traits in males when faced with an intruder [20,21]. Not only does testosterone increase aggressive behaviour in males but aggressive interactions themselves can increase plasma testosterone levels [22]. The association between testosterone and aggression, however, is greatly dependent on the social and ecological context, season, age, experience and taxon [23] and hence is subject to great variability. Steroid hormones have additional roles in the communication context. In some species, testosterone has been shown to increase the bioavailability of carotenoids [24,25], owing to its role in immunosuppression (immunocompetence handicap hypothesis [26]). Additionally, since social interactions can be energy-intensive, glucocorticoid hormones are also expected to influence individual responses. Rise in glucocorticoids during social challenges redirects energy towards immediate needs [27,28]. Glucocorticoids are also known to affect melanin-based coloration [29] by inhibiting melanogenesis [30], and when chronically elevated, can redirect carotenoids from ornaments to immune functions [31].

Since the expression of signals can be affected by any or all of the above factors, understanding the relationship between multiple signals and their underlying physiology remains a challenge. In this study, we determined patterns of signal associations using the tropical agamid lizard Indian rock agama (*Psammophilus dorsalis*). During social interactions, males of this species undergo dynamic colour change [32] on their dorsal and lateral body regions, which contain carotenoid- and pterin-based red, orange and yellow patches [33], melanin-infused brown and black, as well as ultraviolet patches. Males can change their skin colour within seconds of exposure to a conspecific male or female [32]. When interacting with other males, the rapid colour change is accompanied by display behaviours that are directed towards the competitor [32,34,35]. However, the interaction or integration of these multiple signalling traits and whether they influence receiver responses remains unclear. Previous studies have also shown that social interactions in males of *P. dorsalis* can elicit an increase in testosterone and corticosterone levels, which stay elevated for 10−30 min after the interaction [35]. In this study, we explored the patterns of associations between display colours and behaviours in males of *P. dorsalis*. Patterns of associations between trait components can indicate the degree of signal redundancy. We also examined the influence of interaction-induced testosterone and corticosterone levels and body size on display colours and behaviours to identify potential mechanisms involved in the expression of signals. Finally, we assessed whether the expression of each of these signal components is influenced by the receiver’s responses. Overall, our study provides insights on the potential functions and mechanisms shaping the expression of dynamic multi-component signals.

## 2. Methods

### (a) Field capture and housing

Adult male lizards (*n* = 55) were caught from the wild by lassoing, during the months of June to September 2021, from four study sites on the outskirts of Bangalore that were 5−10 km apart. After capture, lizards were placed in separate cotton bags and transported in a cooler lined with icepacks to the lab, where they were housed individually in glass terraria (60 × 30 × 25 cm). Terrariums were lined with paper towels and provisioned with rocks and a shelter for basking and refuge. Terrariums were maintained in a dedicated lizard housing room with automated 12 h light/dark cycle and maintained at ambient temperature conditions. Individual terrariums were also set up with incandescent basking lamps (60 W) that were turned on for 5 h during the day. Lizards were provided with mealworms and grasshoppers daily for food, along with ad libitum water. All lizards were allowed to acclimate for 3 days to laboratory conditions before the start of trials and were maintained in the lab for 9−10 days for the experiments, following which they were released at their site of capture. Ethics clearance for this project was obtained from the institute animal ethics committee (CAF/Ethics/740/2020).

### (b) Blood sampling and hormone analyses

We collected two blood samples from every individual. To determine baseline circulating hormone levels, *ca* 50 µl of blood was drawn from the retro-orbital sinus of each lizard using a heparinized microcapillary tube [35] after 3 days of acclimation in the laboratory. Lizards were allowed 2 days to recover from the first blood sample, before behavioural trials began. To measure social interaction-induced hormone levels, a second sample of *ca* 50 µl of blood was drawn immediately after the end of the staged behavioural trials (see below). Immediately after collection, blood samples were centrifuged and the plasma stored at −20°C, until hormone analysis. Enzyme-immuno assay kits (Arbor Assay DetectX Corticosterone K014-H5; Testosterone K032-H5), optimized for the species [35], were used to measure concentrations of circulating testosterone and corticosterone in the plasma. We analysed plasma at a dilution ratio of 1 : 100 or 1 : 80 for corticosterone and 1 : 140 or 1 : 160 for testosterone. For both hormones, samples were run in triplicates and a total of 13 assays (plates) were run, with a duplicate lab standard in each plate. For corticosterone, mean intra-assay coefficient of variation was 8.08 (ranging from 0.07 to 17.6) and inter-assay coefficient of variation was 15.78. For testosterone, mean intra-assay coefficient of variation was 8.38 (ranging from 1.05 to 18.1) and inter-assay coefficient of variation was 15.07. All blood samples were obtained between 71 and 431 s from the end of social interactions, and there was no significant effect of blood sampling time on any of the hormone levels (*p* < 0.05 for all comparisons). Three individuals in the social interaction trials that had extremely high testosterone levels (>2000 ng ml^−1^) were excluded from all further statistical analyses.

### (c) Social interaction trials

We measured the snout–vent length (SVL) of all lizards (*n* = 46) to ensure they were size-matched for the social interaction trials. Pairs (*n* = 23) were formed by selecting individuals from different field sites, minimizing the likelihood of prior familiarity. Each male lizard was thus paired with an unfamiliar counterpart of similar SVL (±3 mm). Trials were staged in a large glass terrarium (61 cm length × 46 cm width × 32 cm height) in the lab. Lizards were introduced into the testing terrarium on either side of an opaque divider and allowed 60 min to habituate. Once the divider separating them was removed, the pair was allowed to interact for 30 min.

The experimental area was lit using four fluorescent tube lights on the ceiling of the room, one UV tube light (39 W, Reptisun 10.0 UVB T5, Zoo Med Laboratories) 50 cm from the base of the terrarium and a full spectrum bulb (VivaLite: B22) 70 cm from the base of the terrarium (electronic supplementary material, figure S1). The entire experiment arena was covered on all sides by opaque blinds, to minimize disturbance to the lizards during social interaction trials (electronic supplementary material, figure S1).

All trials were simultaneously photographed and recorded on video using two digital cameras, positioned to have an unobstructed angular view of the lizards from above (electronic supplementary material, figure S1). To quantify behaviour, digital videos were recorded using a Cannon 700D camera with an 18−135 mm lens. To obtain images of body coloration, we took digital photographs of each lizard alternately in the UV and visible ranges using a modified multi-spectral Nikon D5500 camera, such that on average photographs were captured at 4 min intervals. This camera was mounted on a metal slider supported by two tripods (electronic supplementary material, figure S1), allowing smooth horizontal and angular adjustments (within a 10° range, along one direction only) as the lizards moved within the terrarium. The multi-spectral camera was fitted with a Nikkor EL 80 mm lens, and a filter wheel (Astromania 2″ 5-Position) containing a 250−390 nm filter (X-Nite 330C) for the UV images and a 400−750 nm filter (X-Nite CC2) for visible-only images (electronic supplementary material, figure S1). The filter wheel allowed quick filter changes between photographs with minimal disturbance to the lizards. The placement of the cameras allowed the lizards’ position to be tracked from behind the blinds (electronic supplementary material, figure S1) and thus, between each photograph, adjustments to the camera position, lens focus and filters were made as needed.

Since the experiment was conducted in a controlled indoor lighting environment, a photograph of a colour checker (X-rite: MSCCPP) placed inside the terrarium was taken under the same lighting conditions at the end of the trials. This image was used to standardize the colours in the visible range. Since only UV patch size was measured, and not the intensity of UV, a UV standard was not included in the terrarium. To confirm that changes in behaviours and colours of lizards were a consequence of social interactions, we ran control trials (*n* = 9) with a single lizard in the experiment terrarium, keeping all other conditions constant (similar to trials run in [32]). All control and social interactions trials, along with blood sampling for hormone analysis (see below), took place between 9.00 am and 12:30 pm, which coincides with the peak activity hours of *P. dorsalis*.

### (d) Social behaviour

From the digital recordings of the 30 min social interactions, we quantified behaviours displayed by both the males of an interacting pair (similar to [35]. In brief, we counted all occurrences of headbobs, shudders, pushups, approaches, attacks and bite behaviours. These behaviours are well characterized and have been previously defined by [35]. All behaviours were measured as events (i.e. count).

### (e) Skin colour measurement

**Figure 1.**
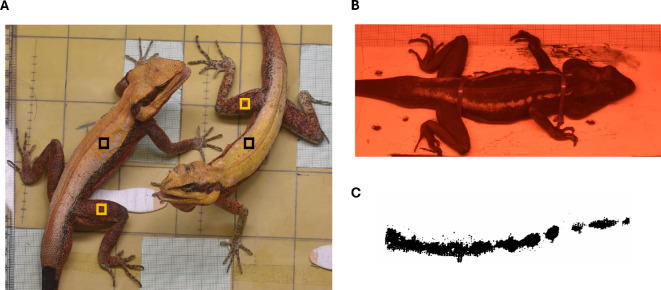
(A) Staged male–male interaction in *Psammophilus dorsalis,* where the black square represents the region of dorsal colour and yellow square represents the region of lateral colour measurement from both the interacting lizards from digital images. (B) UV photograph of a restrained male taken after 10 min of stress handling. (C) UV reflective patch extracted from the UV images using the threshold tool in ImageJ. For both (B) and (C), UV patches are shown for the right side of the lizard only.

We had 6−10 digital images in the visible-only range of each lizard during the trials. Skin colour was quantified from these images as chromatic contrasts measured in terms of just-noticeable differences (JNDs), using the MicaToolBox plugin in ImageJ [36]. All images were first linearized using five grey-scale reflectance squares on a standard colour checker (X-rite: MSCCPP) and a multispectral image was created. In the linearized multispectral image, we created a 2 × 2 mm square region of interest (ROI) on each of the dorsal and the lateral body regions of the lizard (figure 1A). Using the inbuilt chameleon eye model (with cone ratios set as lw:mw:sw = 0.06 : 0.08 : 0.15) and daylight (D65) lighting in the MicaToolBox, we extracted the cone catch values for these ROIs. The cone catch values were then converted to the receptor noise limited (RNL) colour space, and chromatic contrasts were calculated against a standard 40% grey background. We chose a 40% grey background to calculate chromatic contrasts because *P. dorsalis* occupies a variety of substrates in the wild, and the spectral reflectance of the 40% grey falls within the overall reflectance range of these natural substrates (electronic supplementary material, figure S2, modified from [37]). The maximum chromatic contrast on both dorsal and lateral body regions displayed by each individual during the 30 min interaction period was considered for further analysis.

### (f) UV patch size measurement

The size of UV patches was not measured from images taken during the trials because lizards were free to move and so the angle of their bodies with respect to the camera varied across the trial. Therefore, these images did not provide reliable estimates of patch size. Instead, 2 days after the behavioural trials, we induced a stress response in males by restraining each lizard for 10 min, because UV patch size measured under restraint stress can serve as a reliable proxy for the patch size displayed during aggressive interactions [37]. In male lizards, UV patches are positioned along two parallel bands on the dorsal region (figure 1B), and data from previous research [37] as well as the current study (see below) show that the chromatic contrast of the dorsal region expressed during stress handling is positively correlated to that displayed during aggressive interactions (Pearson’s correlation test: dorsal yellow: *R* = 0.7, *p* << 0.01) (electronic supplementary material, figure S3). To obtain images of UV reflective patches, we took photographs of each lizard from both left- and right-side flanks separately, using the Nikon D5500 multi-spectral camera with a 250−390 nm filter on the lens, with the camera positioned perpendicular to the UV patch. The patch areas measured from both the left- and right-side images were summed to determine the total UV patch size for each lizard. A graph sheet was included in each image to provide a scale reference.

We measured the area of the UV patches on the lizard body using the image segmentation technique in ImageJ v1.53K. We employed a protocol for data acquisition slightly modified from that used for images in [38] and [39]. The UV patches were separated from the rest of the lizard body using the image segmentation tool ‘threshold’, with manual thresholding under the ‘Shanbhag’ mode (figure 1C). Because individuals differ in the intensity of UV patch brightness and the size and shape of the UV patches on their body, manual thresholding was used to avoid over- or under-estimation of the UV-covered area. The images were then cleaned using the paintbrush tool to remove any false patches (such as shiny scales or shed skin) that may appear as UV regions. Following thresholding and cleaning, an ROI was created, and the total area of interest was measured by using the ‘Analyse Particles’ tool.

### (g) Statistical analyses

We first tested the normality of every measured trait using the Shapiro–Wilk test and accordingly used either parametric (for chromatic contrast of dorsal yellow) or non-parametric (for chromatic contrast of lateral orange, baseline and interaction-induced testosterone and corticosterone levels) tests for further comparisons of social trials with control (R package: stats).

To confirm that social interactions induced a change in behaviour, colour and hormone levels, we compared the responses of males in control trials to those in social interactions. Males in control trials did not display headbobs and shudders and so no statistical comparisons were made with males in social interaction trials. The maximum chromatic contrast of the dorsal yellow region was compared using Welch’s two-sample *t*‐test and the lateral orange region was compared using the Wilcoxon rank sum test (R package: stats). Hormone levels of control and social-interaction males were compared using Wilcoxon rank sum tests. For the social trials, we also tested the difference between baseline and interaction-induced hormonal responses using a Wilcoxon paired-sample test (R package: stats).

To identify associations among behavioural and colour responses and to derive fewer composite signalling traits, we performed a principal component analysis (PCA) with the number of headbobs and shudders, UV patch size and the maximum chromatic contrast of dorsal and lateral colours (R package: factoextra). To examine whether steroid hormones influenced signalling traits during social interactions, we performed two linear regression models using PC1 and PC2 as response variables, interaction-induced testosterone and corticosterone as predictors, and SVL as a covariate (R package: stats). Since the social trials were run as dyads and traits were measured from both individuals in the dyad, it is possible that the expression of traits is not independent. To address this issue, we bootstrapped both the PCA and linear regression 100 times, subsampling the data to randomly include only one individual from each pair in each iteration (R packages: stats, dplyr).

To determine whether the expression of behavioural and colour traits by individuals was influenced by the responses of their opponents, we calculated the mean difference for each trait between the paired individuals. We then compared this observed difference against a null model distribution, which was generated by calculating the mean difference for each trait across 1000 permutations, with pairs selected randomly (R package: base).

We performed all statistical analyses in R (version 4.4.1 [40]). Codes used for all the analyses are available in Dryad [41].

## 3. Results

### (a) Behavioural responses

**Figure 2.**
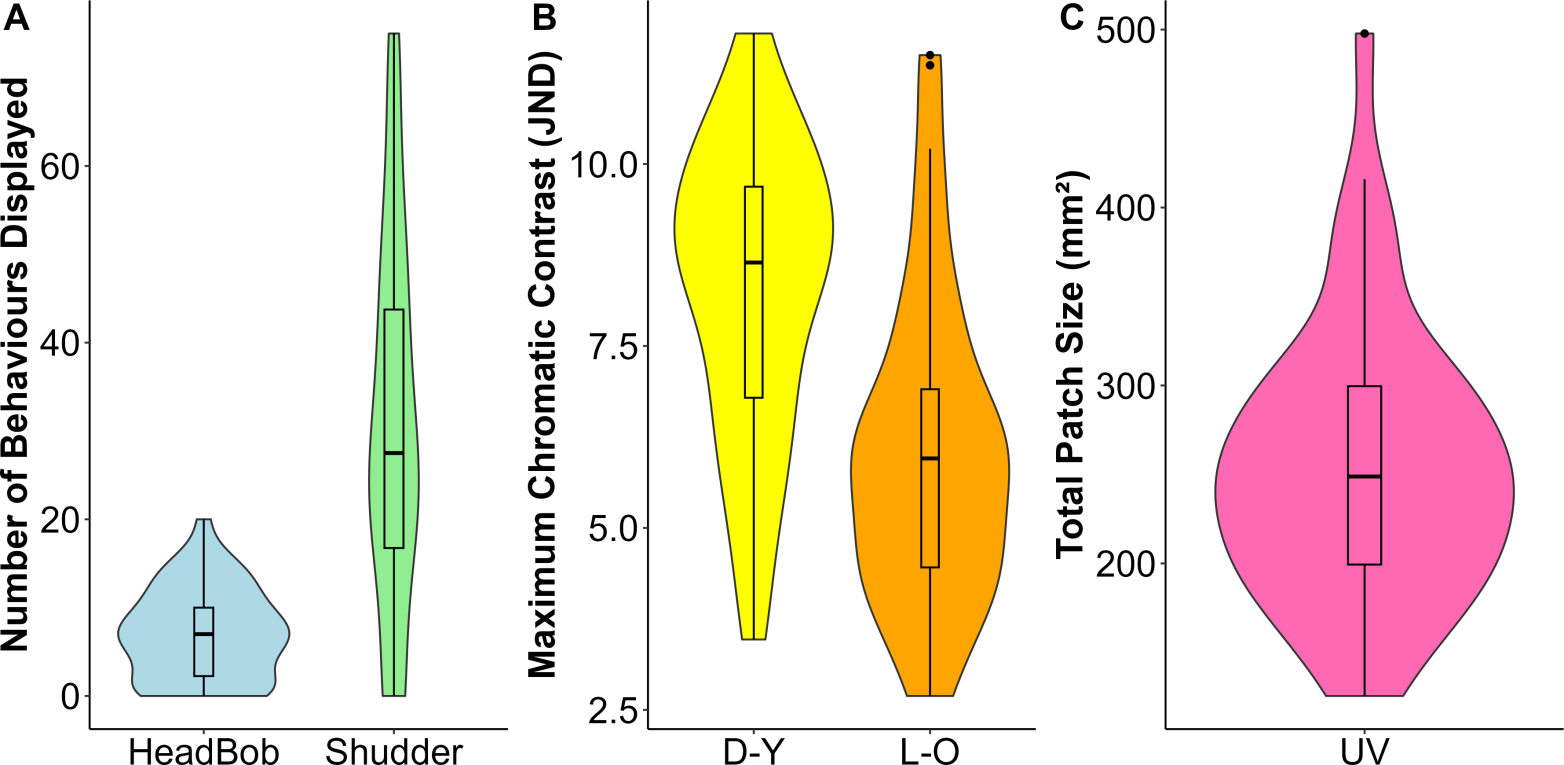
(A) Social behaviours, (B) chromatic contrast of the dorsal yellow (D-Y) and lateral orange (L-O) body regions, and (C) UV patch size, displayed by *Psammophilus dorsalis* males during staged male–male interactions. JND, just-noticeable difference.

During staged male–male encounters, 40 out of 46 animals displayed at least one behaviour. In every trial that elicited a behavioural response, the encounter started with a headbob or shudder and then the pair of males aligned themselves parallel to each other. The encounter then escalated into an attack and bite (39% and 15% of the trials, respectively) or more vigorous head bobs (46%). Out of 46 individuals, 39 exhibited headbobs, 40 displayed shudders, 20 performed pushups, 27 approached their opponent, 18 attacked and 7 showed bite behaviour. Thus, for subsequent analyses, we only retained the headbob and shudder responses as these were the most common responses during an interaction (figure 2A). Two individuals that displayed an unusually high number of atypical shudder displays were excluded from the statistical analyses.

### (b) Physiological colour change

All animals displayed some dynamic colour change and expressed UV reflective patches during the interaction (figure 2B,C). Compared with control trials (no social interaction), the maximum chromatic contrasts displayed by animals in the social interaction trials were higher in both dorsal (Welch two-sample *t*‐test: *t* = 2.46, *p* = 0.03) and lateral body regions (Wilcoxon rank sum test: *W* = 306, *p* = 0.02).

### (c) Hormonal responses

As expected, testosterone and corticosterone levels at baseline were similar for all males, regardless of whether they were in the control (no social interaction) or social interaction groups (Wilcoxon rank sum test: *W* = 233, *p* = 0.28 for testosterone; *W* = 250, *p* = 0.28 for corticosterone) (electronic supplementary material, figure S4).

Following the 30 min social interaction, corticosterone levels increased significantly in both control and social interaction groups compared with baseline levels (Wilcoxon paired sample test: social males: *V* = 293, *p* = 0.01; control males: *V* = 5, *p* = 0.04). Contrary to expectation, testosterone levels did not increase after the 30 min social interaction trials compared with baseline (*V* = 356, *p* = 0.24). Testosterone levels also did not change significantly in the control males after 30 min in the tank, compared with baseline (*V* = 29, *p* = 0.5) (electronic supplementary material, figure S4).

Finally, there was no significant difference between control and social interaction males in their testosterone and corticosterone levels after the 30 min trials (Wilcoxon rank sum test: *W* = 250, *p* = 0.14 for testosterone; *W* = 215, *p* = 0.86 for corticosterone) (electronic supplementary material, figure S4).

### (d) Associations within signalling traits

**Figure 3.**
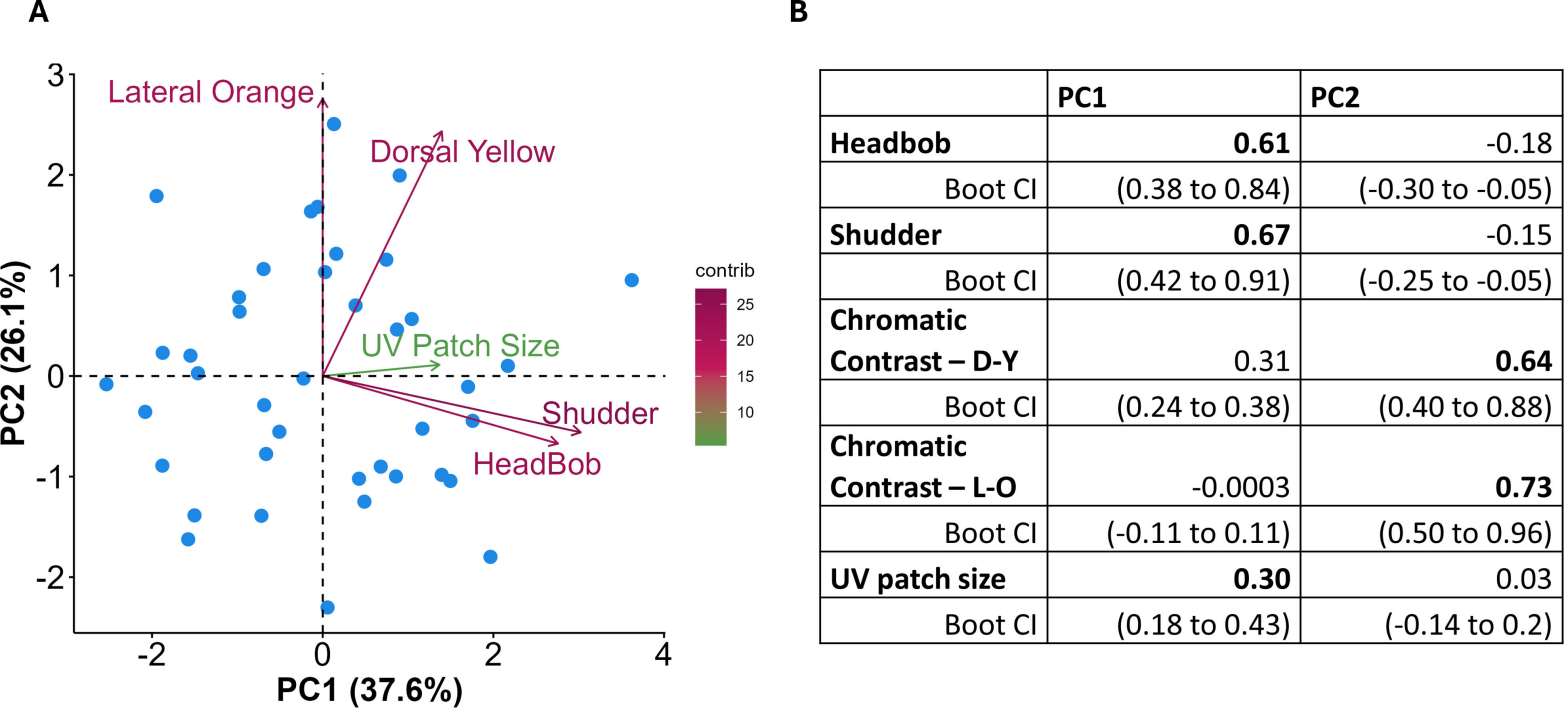
(A) Principal component (PC) analysis of behaviours (number of headbobs, shudders) and colours (maximum chromatic contrast of dorsal yellow (D-Y), lateral orange (L-O) and UV patch size) displayed by *Psammophilus dorsalis* males during staged male–male interactions. (B) PC loadings and their corresponding bootstrapped confidence intervals (Boot CI) for the signalling traits.

The PCA analysis yielded PC1 (37.6% of the variance in the data) and PC2 (26.1%), which represent most of the variance in the data (figure 3A; Bartlett’s test of sphericity: *χ*^2^ = 41.45, *p* < 0.001). Number of headbobs, number of shudders and UV patch size loaded positively on PC1, with loadings ranging from 0.30 to 0.67 (figure 3B). The chromatic contrasts of the dorsal yellow (0.64) and lateral orange (0.73) regions loaded positively on PC2 (figure 3B). These patterns remained consistent after bootstrapping the data (figure 3B). Six individuals with missing trait values were removed from the PCA analysis.

### (e) Influence of steroid hormones and body size on signalling traits

**Figure 4.**
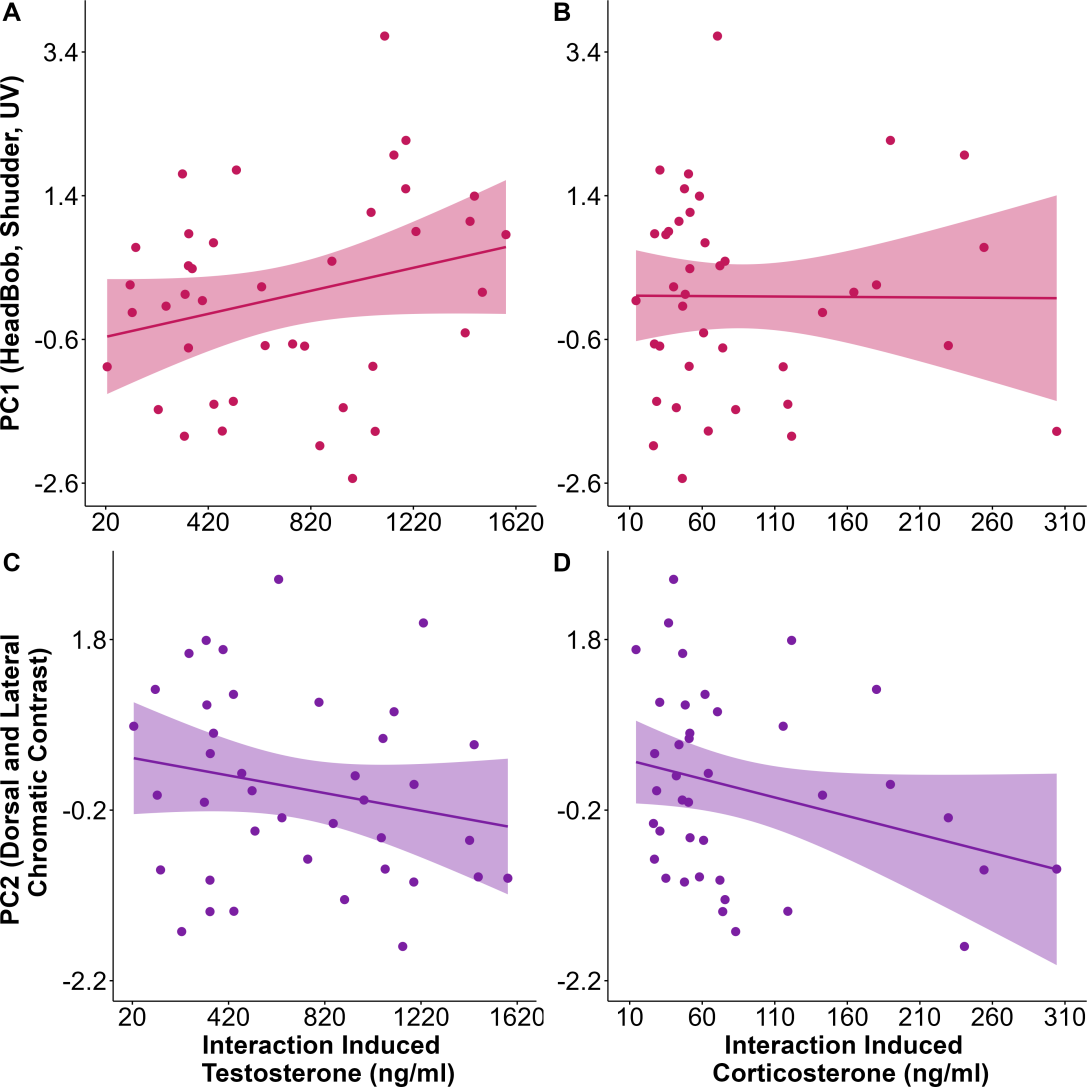
Plots showing association between interaction-induced hormone levels and social signals. Behaviours and UV patch sizes (PC1) displayed by *Psammophilus dorsalis* males are positively associated with testosterone (A) but not corticosterone (B) levels. Similarly, the maximum chromatic contrasts expressed on the dorsal and lateral body region (PC2) are negatively associated with both testosterone (C) and corticosterone (D) levels.

Both testosterone (*t* = −1.88, *p* = 0.06) and corticosterone (*t* = −2, *p* = 0.05) had a negative effect on PC2 values (primarily the maximum chromatic contrast of the dorsal and lateral body regions) (figure 4C,D). In contrast, PC1 values (i.e. headbob, shudder and UV patch size) were positively but weakly influenced by testosterone levels (*t* = 1.69, *p* = 0.09; figure 4A) and not affected by corticosterone levels (*t* = 0.18, *p* = 0.86; figure 4B). These patterns of associations remained consistent even after 100 iterations of random subsampling and bootstrapping the data. Model coefficients and their bootstrapped 95% confidence intervals are shown in table 1. Body size had no significant influence on any of the signalling traits (PC1: *t* = 0.69, *p* = 0.49; PC2: *t* = 1.04, *p* = 0.31).

**Table 1.** Effect of testosterone and corticosterone on the signalling traits (PC1 and PC2) displayed by *Psammophilus dorsalis* during staged male–male interactions. Model coefficients from the linear regression, bootstrapped confidence intervals for coefficients and *p*-values are shown.

	coefficient	bootstrapped 95% confidence interval	***p*-value**
*PC1 (headbob, shudder, UV)*			
interaction-induced testosterone	0.0008	0.0006 to 0.001	0.09
interaction-induced corticosterone	0.0005	−0.0006 to 0.002	0.85
*PC2 (dorsal and lateral chromatic contrast)*			
interaction-induced testosterone	−0.0007	−0.0008 to −0.0006	0.06
interaction-induced corticosterone	−0.005	−0.006 to −0.004	0.05

### (f) Opponent response

**Figure 5.**
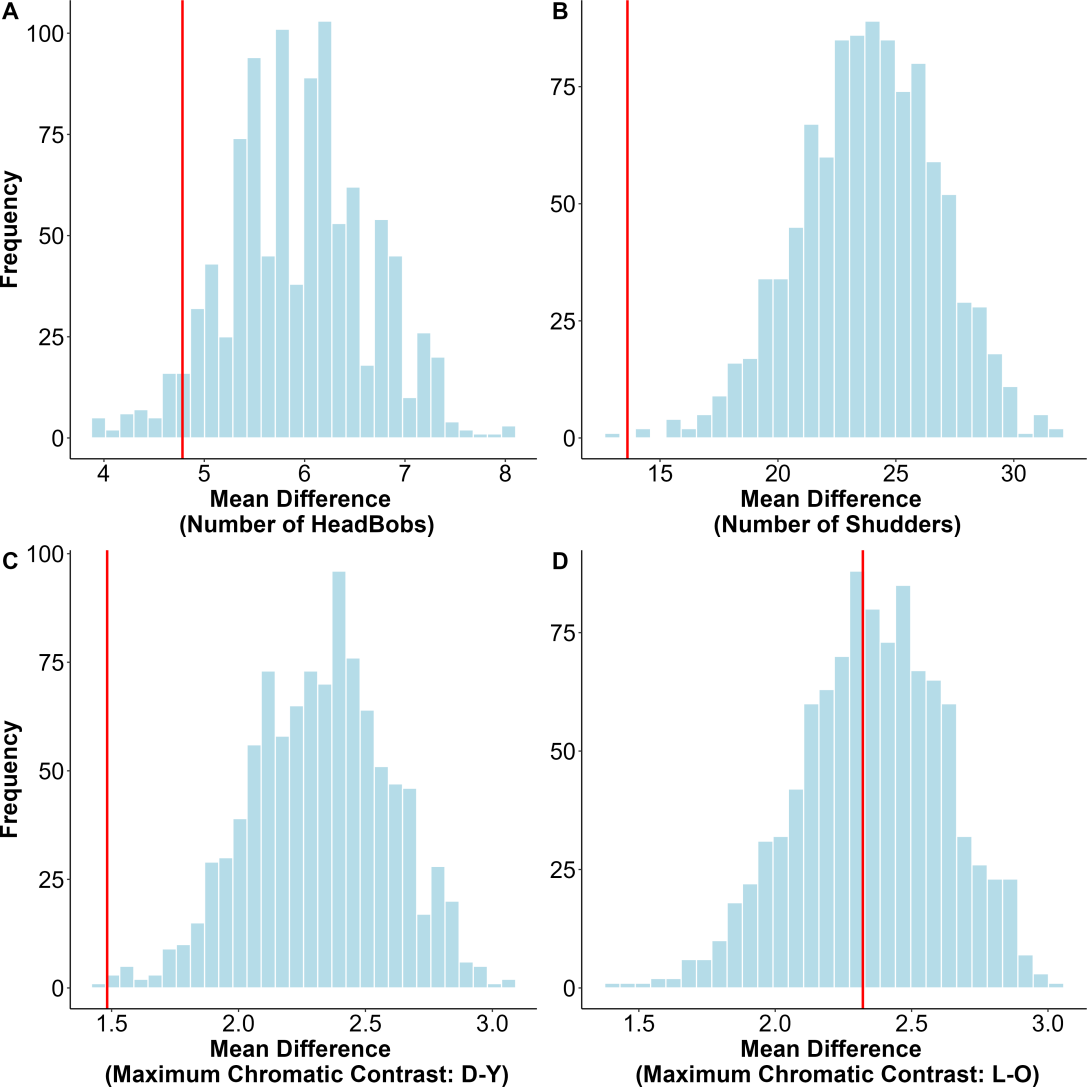
Observed mean difference (represented by the red vertical line) between pairs of *Psammophilus dorsalis* males, modelled against a null distribution for each of the signalling traits displayed during social interactions: (A) number of headbobs, (B) number of shudders, (C) maximum chromatic contrast of dorsal yellow (D-Y), and (D) maximum chromatic contrast of the lateral orange (L-O). The null distribution represents the mean differences in trait values when pairs are selected at random.

During social interactions, individuals within a pair closely matched their display behaviours (observed mean difference against null distribution: *p* = 0.06 for headbobs, *p* = 0.002 for shudders). Males also closely matched the maximum chromatic contrast of the dorsal yellow colour (*p* = 0.005) with their opponents (figure 5A–C). However, the maximum chromatic contrast of the lateral orange colour was not matched between pairs (*p* = 0.43) (figure 5D).

## 4. Discussion

A complex signalling system is generally composed of multiple components that either convey redundant information or encode multiple messages. Traits that are structurally or physiologically similar might share pathways of expression, leading to redundancy. Therefore, components of a signalling system can be coupled (redundant, shared pathway) or decoupled (multiple messages, no shared pathway) depending on their function or mechanisms of expression. Additionally, the expression of signalling traits and the associations between them can be actively modulated by physiological drivers such as steroid hormones or contextual factors such as receiver responses. In *P. dorsalis*, we find that social interactions between males involve the expression of multiple behavioural and colour displays, and an elevation of corticosterone but not testosterone. Similar traits within the categories of colour and behaviour were closely associated, with variation in certain signalling traits better explained by mechanistic drivers such as steroid hormones but not body size. Receiver response also seems to play a role in the expression of these traits during social interactions.

Signals that contain the same information (i.e. are redundant) or that share the same pathway of expression are expected to be strongly correlated. In *P. dorsalis*, multiple behavioural displays were positively associated. Animals that displayed more headbobs were also likely to display more shudders. Such variation in overall behavioural activity may indicate inherent differences among males in the level of aggression that they express during male–male interactions, consistent with observations in other lizard species [42,43]. Similarly, the maximum chromatic contrasts of the dorsal yellow and lateral orange regions expressed during the social interactions were also positively associated, suggesting that both these colours could potentially encode redundant information regarding individual quality. Shared physiological pathways could also explain the correlation of behaviours within individuals, as the expression and intensity of behaviours are largely dependent on the musculoskeletal endurance of the limbs and neck as well as motor capabilities [19,44]. Among the colours expressed, both the dorsal yellow and lateral orange colours in *P. dorsalis* are composed of multiple pterins and carotenoid pigments [33], and thus a shared biochemical pathway may explain their close association. Additionally, UV patch sizes were positively associated with intensity of display behaviours, such that behaviourally reactive individuals had larger UV patch sizes. This pattern is consistent with findings from studies on other lizard species, where UV colour patches are associated with aggression or dominance [45,46]. Given that UV patches are dynamically expressed during stress and aggression in *P. dorsalis*, males may be able to reliably assess each other using the size of UV patches.

Signals that show little or no association can potentially convey multiple messages. In *P. dorsalis*, display behaviours were not associated with the maximum chromatic contrast of body colours, suggesting that these traits might encode distinct information regarding individual quality. Conspicuous colours and behaviours, which are easily discernible by receivers but costly to produce and maintain, are often indicators of individual quality. Numerous studies on birds, fish and reptiles have reported the usage of carotenoid, UV and melanin colours to signal aggression during social contests [47,48]. However, the specific information conveyed by these signal components in *P. dorsalis* remains unclear. Notably, previous studies have shown that the chromatic contrasts of dorso-lateral body regions in males are not associated with sprint speed or bite force [33]. Furthermore, the lack of association between UV patch size and the chromatic contrast of the dorso-lateral body region indicates that the expression of pigment-based and structural-UV colours likely operate through separate pathways or convey non-redundant information.

In many vertebrates, testosterone plays a role in modulating the expression of signals, such as colours and behavioural displays, during social interactions (22). For androgens to directly modulate social behaviours or dynamic colour expression, we would expect individuals to show an increase in testosterone levels during social encounters (i.e. challenge hypothesis [21]). However, social interactions in *P. dorsalis* did not elicit an increased testosterone response in our study, which is similar to findings in several other species of birds, fish and lizards [49,50]. Nevertheless, testosterone levels expressed during social interactions were positively related to the intensity of display behaviours and UV patch size (albeit weakly) and negatively with chromatic contrast of dorso-lateral colours. The lack of a rise in testosterone above breeding baseline levels in response to a social challenge, along with its weak association with display behaviours, could be explained by the timing of the experiments. Conducted during the peak breeding season, these experiments likely involved animals already engaged in breeding-related social interactions prior to capture. It is possible that testosterone levels were already sufficiently elevated to facilitate an aggressive response, a pattern observed in other tropical species [51]. Moreover, as the breeding season was already underway, male lizards had likely established territories with less frequent aggressive male–male interactions. Therefore, the responsiveness of testosterone to male–male encounters may be reduced at this stage of the breeding season [21,23]. Although the negative association between testosterone level and chromatic contrast of colours is counterintuitive (see immunocompetence handicap hypothesis [26]), these findings corroborate results from previous studies in the same species, which identified a negative association between testosterone level and dorsal yellow chromatic contrast [33].

Following social interactions in *P. dorsalis,* we detected a rise in plasma corticosterone from baseline levels. Animals with higher corticosterone levels during social interactions exhibited lower chromatic contrast on their dorso-lateral body regions but the intensity of display behaviours and UV patch size were unaffected. Although evidence linking glucocorticoids to ornamentation is mixed [52,53], our findings support multiple studies in birds, reptiles and amphibians that have found negative associations between glucocorticoid levels and carotenoid-based colours [31,54,55]. Although we expected corticosterone levels to influence aggressive behaviour directly [56], we find no support for this in our data. Since males that did not engage in a social interaction (control individuals) also showed a rise in corticosterone from baseline levels over the same duration, it is possible that 30 min in a novel environment elicits a stress response regardless of the additional social encounter. Alternatively, corticosterone could also have a permissive role, where its presence is necessary only for expression but not modulation of aggressive behaviour [28].

In addition to hormonal influences, morphological traits such as body size can also influence the expression of display traits by acting either as a signal itself or as a mechanistic mediator. Body size is known to provide information to receivers about aggressive behaviour, bite force or dominance [57,58]. Hence, during aggressive interactions, animals tend to assess their opponent’s body size [59,60]. At a mechanistic level, body size is associated with key life history traits as well as physiology, and thus variation in body size can result in corresponding variation in the expression of display traits [61,62]. However, in *P. dorsalis,* body size (SVL) did not have any influence on the display traits. Since all the animals in our experiments were paired with similarly sized opponents, body size would not have provided any additional information as a signal. Additionally, as the experimental lizards fell within a narrow range of body sizes (11.2–13.7 cm), any mechanistic effects of body size on display traits were likely minimal and would have been overshadowed by context-dependent responses from receivers. Under natural conditions, however, males of *P. dorsalis* likely interact with other males of different sizes, and thus body size, as either an absolute or relative measure, could act as an independent signal during social interactions.

Receiver response is widely accepted as one of the major factors that drive signal evolution and maintenance [63]. However, the ability to modulate signal quality leaves ample room for signal manipulation and dishonest signalling [11]. For example, male mourning cuttlefish (*Sepia plangon*) tactically display deceptive signalling patterns to prevent rival males from gaining courtship [64]. Nevertheless, aggressive interactions with receivers are known to impose a cost that ensures signal honesty over evolutionary time [63]. We found that during aggressive interactions with size-matched opponents, male lizards matched the intensity of their behavioural displays and the maximum chromatic contrast of dorsal yellow colour with that of the opponent’s behaviour and colour, respectively. This means that males with high chromaticity of dorsal colour and high behavioural aggression elicited similarly high responses in both these traits in their opponents. The chromatic contrast of the lateral orange colour was not matched by the opponents, suggesting that opponents probably pay less attention to this colour during an interaction. Previous studies have shown that the dorsal yellow colour in male *P. dorsalis* is more conspicuous to predators, such as snakes, dogs and birds, compared with the lateral orange colour [37]. During a fight, when lizards increase the chromatic contrast of their dorsal yellow colour while also being behaviourally active, their conspicuousness to a predator will likely increase, thus increasing their predation risk. Hence, in line with the handicap hypothesis [65], the costs associated with the dorsal colour trait suggest that it may function as a reliable indicator of male quality to conspecifics. Thus, while predation risk enforces a selection pressure on the expression of conspicuous traits, receiver response might reinforce signal honesty in this system, since aggressive individuals elicit more aggression and attacks from opponents, making it difficult for weaker individuals to cheat.

In summary, the signalling system of *P. dorsalis* involves the expression of multiple dynamic traits that vary in their degree of redundancy and their associations with steroid hormone levels. Our study adds to the growing body of evidence that highlights the complexity of animal communication signals and the need for more comprehensive, manipulative studies. We find that complexity in signalling systems may reflect the need for both redundancy and non-redundancy, especially during male–male interactions that have fitness consequences. Notably, the hormonal mechanisms mediating the expression of these signalling traits differ from expected patterns. Overall, our study provides insights on how dynamic multi-component signals are maintained, from a functional as well as a mechanistic perspective.

## Data Availability

All data and code are available at [41]. Supplementary material is available online [66].
